# A LIFESPAN DEVELOPMENTAL ANALYSIS OF RECIPROCAL RELATIONS BETWEEN HEAVY DRINKING AND SLEEP PROBLEMS

**DOI:** 10.1093/geroni/igad104.2868

**Published:** 2023-12-21

**Authors:** Douglas Bowlby

**Affiliations:** Rutgers University, New Brunswick, New Jersey, United States

## Abstract

In recent years, numerous studies have begun to explore the ways in which problem drinking behavior is affected by different lifespan developmental changes. However, while there currently exists a plethora of research on adolescence and young adulthood, there is far less research with midlife and older adults examining lifespan developmental changes and problem drinking. As part of larger NIAAA-funded project investigating aging, health, and alcohol use, the current study investigated cross-sectional and longitudinal associations among heavy drinking and sleep problems in a sample of midlife and older adults. One particularly noteworthy analysis used path analysis in Mplus to test a longitudinal moderated mediation model involving sleep problems, sleep-related motives for drinking, and heavy drinking. Results showed an interaction between sleep problems and sleep-related drinking motives. Interestingly, sleep problems appear to significantly reduce the risk for heavy drinking among most participants. However, when combined with high sleep-related drinking motives, sleep problems appear to significantly increase risk for heavy drinking. This phenomenon may help explain the occasionally contradictory results found in previous studies examining the impact of sleep disturbances on drinking behavior and may prove to be an area worth investigating in future studies. From a lifespan-developmental perspective, these findings highlight how there are likely important differences across stages of adulthood in the most relevant factors that affect heavy drinking. Health-related factors including sleep problems, coupled with motives to use alcohol to cope with such problems, appear to be a pathway to heavy drinking that increases in importance across the adult lifespan.

